# The Key Roles of *Mycobacterium tuberculosis* FadD23 C-terminal Domain in Catalytic Mechanisms

**DOI:** 10.3389/fmicb.2023.1090534

**Published:** 2023-02-21

**Authors:** Mengrong Yan, Lin Cao, Li Zhao, Weihong Zhou, Xiang Liu, Wei Zhang, Zihe Rao

**Affiliations:** ^1^State Key Laboratory of Medicinal Chemical Biology, Frontiers Science Center for Cell Responses, College of Life Sciences, Nankai University, Tianjin, China; ^2^Innovative Center for Pathogen Research, Guangzhou Laboratory, Guangzhou, China; ^3^Shanghai Institute for Advanced Immunochemical Studies and School of Life Sciences and Technology, ShanghaiTech University, Shanghai, China; ^4^Laboratory of Structural Biology, School of Life Sciences and School of Medicine, Tsinghua University, Beijing, China

**Keywords:** sulfolipid-1, fatty acid adenylating enzyme, crystal structure, enzymatic activity, catalytic mechanism

## Abstract

Sulfolipid-1 (SL-1) is located in the *Mycobacterium tuberculosis* (*M. tb*) cell wall, and is essential for pathogen virulence and intracellular growth. Multiple proteins (e.g., Pks2, FadD23, PapA1, and MmpL8) in the SL-1 synthesis pathway can be treated as drug targets, but, to date, their structures have not been solved. The crystal structures of FadD23 bound to ATP or hexadecanoyl adenylate was determined in this study. We have also investigated long-chain saturated fatty acids as biological substrates of FadD23 through structural, biological, and chemical analyses. The mutation at the active site of FadD23 greatly influences enzymatic activity. Meanwhile, the FadD23 N-terminal domain alone cannot bind palmitic acid without C-terminal domain facilitation since it is almost inactive after removing the C-terminal domain. FadD23 is the first protein in the SL-1 synthesis pathway whose structure has been solved. These results reveal the importance of the C-terminal domain in the catalytic mechanism.

## 1. Introduction

Tuberculosis (TB) is a communicable disease that is a major cause of ill health and death worldwide (Global tuberculosis report, 2021). *Mycobacterium tuberculosis* (*M. tb*), the causative agent of TB, has a complex cell wall structure that protects the pathogen from harsh environments, antibiotics, and host factors. Sulfolipid-1 (SL-1), one of the important unique components of the pathogenic *M. tuberculosis* cell wall (Goren et al., 1971; Schelle and Bertozzi, 2006), is a key inhibitor of mitochondrial oxidative phosphorylation, phagosome-lysosome fusion, and induces changes in phagocytic cells (Kato and Goren, 1974; Goren et al., 1987; Zhang et al., 1988). In addition, recent studies have demonstrated that SL-1 is necessary and sufficient to trigger neuronal activation and coughing in humans (Ruhl et al., 2020). Collectively, this shows that SL-1 plays a key role in the pathogenesis of tuberculosis. Hence, many efforts have been directed toward investigating pivotal proteins in the SL-1 biosynthetic pathway and understanding how *M. tuberculosis* regulates this lipid or its precursors as a potential mechanism for host immune regulation to identify new anti-TB drug targets.

**Figure 1:**
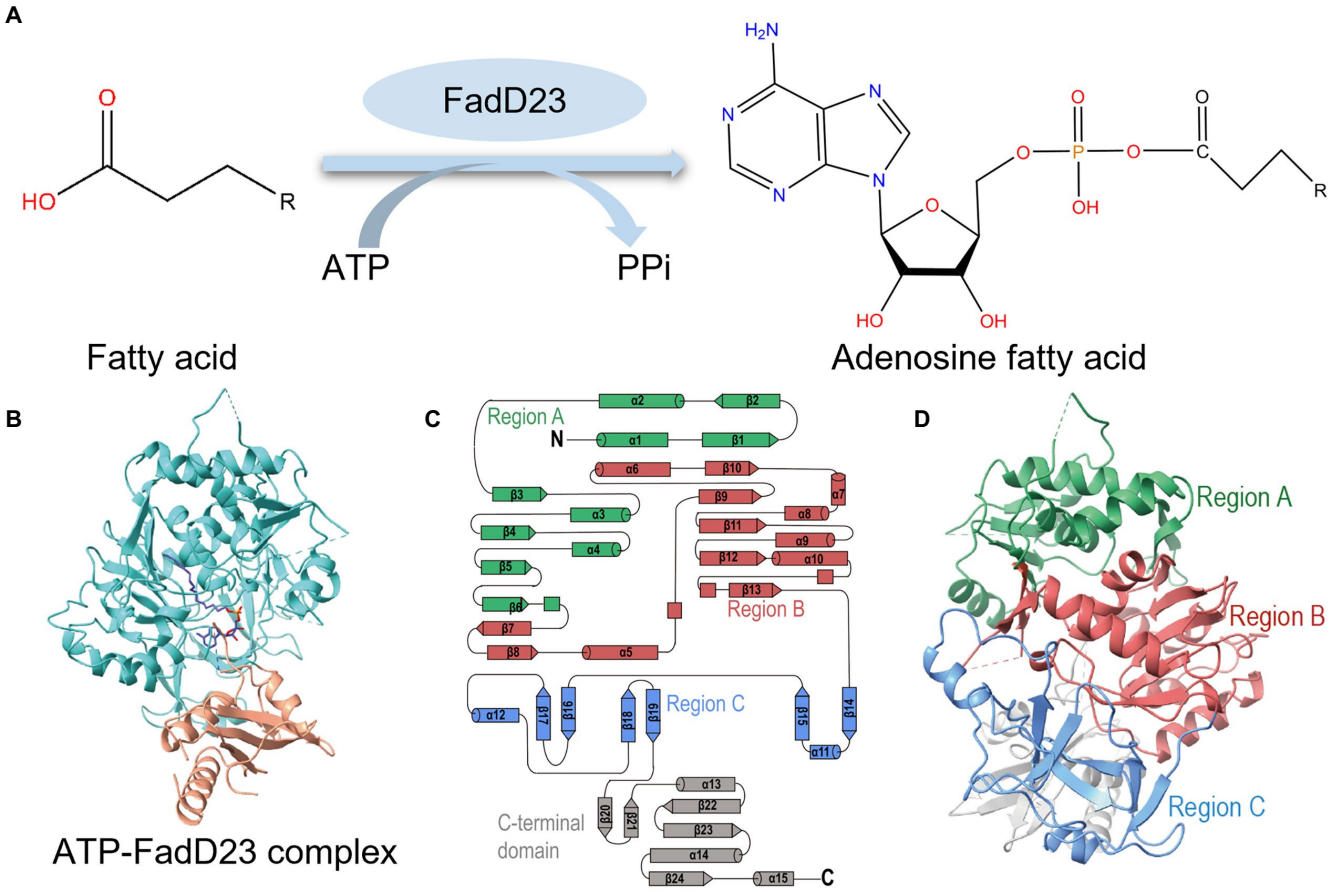
Overall structure of *Mycobacterium tuberculosis* (*M. tb*) FadD23. **(A)** The catalytic scheme of FadD23. FadD23 utilizes fatty acids and activates them for acyl adenylate and PPi production. **(B)** Crystal structure of the ATP-FadD23 complex (N-terminal domain colored in cyan; C-terminal domain colored in tawny; hexadecanoyl adenylate colored in purple). **(C)** Topology diagram. **(D)** The N-terminal domain can be divided into three regions: regions A, B, and C, colored green, red, and blue, respectively.

Sulfolipid-1 consists of one trehalose-2-sulfate (T2S) disaccharide with four acyl groups (Mougous et al., 2004): a straight-chain fatty acid (palmitate or stearate) and three multiple methyl-branched (hydroxy) phthioceranoic acids. As previously described, the first step in SL-1 biosynthesis is the sulfation of trehalose (Tre) by Stf0 to form T2S (Mougous et al., 2004). Subsequently, an acyltransferase, PapA2, adds one straight-chain fatty acid to the 2′-position of T2S to generate SL_659_ (Kumar et al., 2007). Thereafter, another acyltransferase, PapA1, transfers one multiple methyl-branched (hydroxy) phthioceranoic acid to the 3′-position of SL_659_ to form SL_1278_ (Kumar et al., 2007). Additional catalytic steps are acylation at the 6- and 6′-positions of SL_1278_ with two multiple methyl-branched (hydroxy) phthioceranoic acids, most likely through membrane-associated Chp1 to produce fully elaborated SL-1 (Seeliger et al., 2012). These multiple methyl-branched (hydroxy) phthioceranoic acids are synthesized by polyketide synthase 2 (Pks2) using an activated fatty acid starter unit provided by fatty acyl AMP ligase FadD23 (FAAL23; Figure 1A; Sirakova et al., 2001; Gokhale et al., 2007). In addition, the lipid transporter, MmpL8, and integral membrane protein, Sap, are required for biosynthesis and transport (Converse et al., 2003; Seeliger et al., 2012).

FadD23-Pks2 plays a central role in SL-1 synthesis (Lynett and Stokes, 2007). Pks2 is involved in synthesizing such long-chain multiple methyl-branched fatty acids. However, disrupting the gene encoding the enzyme responsible for synthesizing these acids may produce sulfolipid-deficient mutants (Sirakova et al., 2001). FadD23 is one of the 34 fatty acid adenylating enzymes (FadDs) in *M. tuberculosis* (Cole et al., 1998). The first class of FadDs comprises fatty acyl-coenzyme A (CoA) ligases (FACLs) that may be involved in lipid degradation and remodeling (Kuhn et al., 2016). FACLs use CoA as a common substrate to form acyl-CoA. Another class of FadDs comprises FAALs involved in the biosynthesis of toxic lipids in mycobacteria, as well as in the biosynthesis of lipopeptides of pharmaceutical importance in other microorganisms. FAALs activate and transfer fatty acids onto related Pks proteins to further synthesize the diverse *M. tuberculosis* lipids (Mohanty et al., 2011). FAALs use a carboxylate substrate and ATP to form acyl-AMP. It is speculated that FAALs transfer the acyl chain to the Pks modified with the phosphopantetheinyl (P-pant) arm through a special protein–protein interaction (Buchholz et al., 2009; Gavalda et al., 2009). Biochemical test corroborates that acyl-AMP ligases (FadD26, FadD30, and FadD32) can specifically transfer activated fatty acids to the cognate Pkss (Trivedi et al., 2004).

Typically, FadDs adopt two main conformations: adenylation and thiolation. The C-terminal domains undergo a domain alternation of approximately 140° to complete the catalysis steps. The active site is located at the interface of the N- and C-terminal domains (Reger et al., 2007, 2008; Kochan et al., 2009; Goyal et al., 2012). One research group identified 5′-O-[N-(11-phenoxyundecanoyl) sulfamoyl]-adenosine (PhU-AMS) as the optimal ligand for *M. tb* FadD32, and PhU-AMS was shown to inhibit the *M. tb* FadD32-catalyzed loading of Pks13 (Kuhn et al., 2016). Another study showed that 11-phenoxyundecanoyl-AMS has an inhibitory effect on *M. tb* FadD28 (Baran et al., 2020). However, whether non-hydrolyzable analogs of acyl-AMS have an inhibitory effect on other FAALs is still unknown. *Mycobacterium tuberculosis* FadD23 and *M. tb* FadD28 have 66% sequence similarity and share the same evolutionary branch; however, only the structure of the N-terminal domain of *M. tb* FadD28 is available. Here, we solved the full-length *M. tb* FadD23 structure and attempted to elucidate its molecular mechanism.

To gain insight into substrate recognition, as well as to provide a foundation for designing drugs against *M. tuberculosis*, our research identified the 2.68 Å crystal structure of *M. tb* FadD23 complexed with ATP and the 2.25 Å crystal structure with AMP-PNP. We observed palmitic acid in the crystal structure indicating that the protein moiety most likely utilized palmitic acid more than other *Escherichia coli* fatty acids. Meanwhile, FadD23 included a hydrophobic tunnel and has substrate recognition specificity, as verified by biochemical experiments. We also elucidated the 1.5 Å crystal structure of the *M. tb* FadD23 N-terminal domain and verified that the C-terminal domain is essential in lipid synthesis through biochemical experiments. Based on our research and previous reports, we propose a structural basis for the substrate binding and generation of acylation reactions. Furthermore, we propose that FAALs adopt the C-terminal “lid” domain model and exert their enzymatic activity through the conformational change in the C-terminal “lid” domain to generate the product.

## 2. Materials and methods

### 2.1. Cloning, expression, and purification of protein

The *fadD23* gene, encoding the fatty-acid-AMP ligase, FadD23, was amplified from genomic DNA of *M. tuberculosis H37Rv* by PCR. The amplified DNA was then inserted between restriction sites *Nde* I and *Xho* I of a pET15b expression vector with a 6 × Histidine (His) tag at the N-terminus. The resulting plasmid was transformed into *E. coli* BL21(DE3) plySs. Cells were grown in Lysogeny broth (LB) medium with 100 μg/ml ampicillin at 37°C. When the optical density at 600 nm (OD_600_) reached 0.7, the culture was treated with 0.2 mM isopropyl-D-thiogalactopyranoside (IPTG) at 37°C to induce protein expression. To induce FadD23 expression in *Mycobacterium smegmatis* (*M. smeg*) mc^2^155 cells, *fadD23* was sub-cloned into a pMV261 vector with an 8 × His tag at the C-terminus, under the control of an acetamide promoter. The resultant plasmid was transformed into *M. smegmatis* mc^2^155 cells by electroporation. The cells were cultivated in LB liquid media supplemented with 50 μg/ml kanamycin and 20 μg/ml carbenicillin. When the cells were grown to an optical density (OD_600_) of 1.0–1.2 at 37°C, overexpression of the recombinant protein was induced by 0.2% (w/v) acetamide at 16°C. Supplementary Table S1 lists the primers used in this study.

*Escherichia coli* and *M. smegmatis* mc^2^155 cells were then harvested within 5 and 96 h of induction, respectively, and resuspended in lysis buffer (50 mM Tris·HCl, pH 8.0; 300 mM NaCl; and 5% glycol). Thereafter, cell debris was removed by high-speed centrifugation at 18,000 rpm for 40 min after crushing using a homogenizer (AH-Basic-II, ATS Engineering Limited, China). Next, the supernatant was loaded onto an Ni-NTA affinity column (Cytiva, Marlborough, MA, United States) and washed in the resuspension buffer (50 mM Tris·HCl, pH 8.0; 300 mM NaCl; 5% glycol; and 20 mM imidazole). Subsequently, the protein was eluted in elution buffer (50 mM Tris·HCl, pH 8.0; 300 mM NaCl; 5% glycol; and 300 mM imidazole). FadD23 was further purified by ion-exchange and size-exclusion chromatography (Supplementary Figure S1A). Finally, the protein was stored in 20 mM Hepes, pH 7.5, and 150 mM NaCl. The construction and purification methods of the FadD23 N-terminal domain (1–465 AA) were the same as those of the full length.

### 2.2. Crystallization

Apo-FadD23 only yielded poor-quality crystals that did not produce usable diffraction data. Holo-FadD23 produced crystals suitable for diffraction experiments. 1 μl FadD23 (8 mg/ml), containing 2 mM ATP and 2 mM MgCl_2_, was mixed with 1 μl well solution containing 140 mM sodium citrate tribasic dihydrate, 60 mM magnesium chloride hexahydrate, 30 mM tris hydrochloride (pH 8.5), 14% (w/v) polyethylene glycol 3,350, and 9% (w/v) polyethylene glycol 4,000. The crystal of the AMP-PNP-FadD23 complex suitable for diffraction experiments contained 70 mM Tris (pH 8.5), 140 mM sodium citrate tribasic dihydrate, 140 mM ammonium phosphate monobasic, 35% w/v (+/−)-2-methyl-2,4-pentanediol, and 14% (w/v) polyethylene glycol 3,350. The crystal of the FadD23 N-terminal domain suitable for diffraction experiments contained 70 mM Bis-Tris pH 6.5, 60 mM sodium chloride, 20% (w/v) polyethylene glycol 3,350, and 6% (w/v) polyethylene glycol 6,000. Single crystals were carefully harvested from clustered crystals and were soaked in a well solution supplemented with 20% glycol for 10 s before been frozen in liquid nitrogen.

### 2.3. Data collection, processing, and structure determination

X-ray data were collected from beamline BL17U1, BL18U1, and BL19U1 at the Shanghai Synchrotron Radiation Facility (SSRF) at 100 K. Data integration and scaling were performed using XDSgui (Kursula, 2004; Kabsch, 2010). The structure was determined by molecular replacement using the Phaser module in Phenix (McCoy et al., 2007; Adams et al., 2010) using the *M. tb* FadD28 [RCSB Protein Data Bank code (PDB: 3E53)] N-terminal as a search template. The output model of the molecular replacement subsequently built the C-terminal using electron density and was subjected to iterative cycles of manual model adjustment using Coot (Emsley and Cowtan, 2004). Refinement was conducted using Phenix. The processing methods of different crystal structure were similarly conducted.

### 2.4. AlphaFold2 predictions

The full AlphaFold v2.0 (AlphaFold2) pipeline was obtained from DeepMind and installed onto a local workstation (Jumper et al., 2021). Structure predictions of full-length FadD23 were performed using AlphaFold2 software. The Local Distance Difference Test (pLDDT) plot was constructed using AlphaPickle (Arnold, 2021).

### 2.5. Mass spectrometer analysis

The mass spectrometer (MS) analysis samples were: 200 μl of purified FadD23 (1 mg/ml) or buffer extract fatty acid using 600 μl of 100% trichloromethane. Then, the solution was dried by Termovap Sample Concentrator (N-EVAP-24, Organomation, United States), and dissolve in 100% acetonitrile. The MS analysis of the FadD23 sample and unknown fatty acids was performed using exact mass TOF-MS (Xevo G2-XS Tof, Waters, United States) *via* an electrospray ionization source mass spectra were recorded in the negative ionization mode over a 50–1,200 m/z mass range. The scan parameters included 2.5 kV capillary voltages and 1 s scan times. Other source conditions were as follows: 100°C source temperature, 50 and 600 l/h cone, and desolvation gas flow, respectively, and 40 V cone voltage. Data processing and analysis were performed using MassLynx V4.1 (Waters, United States).

### 2.6. Differential scanning fluorimetry

The Differential scanning fluorimetry DSF method is a widely used for determining protein stability. When a ligand is bound to the protein, the stability of the protein increases, such that the exposed hydrophobic groups at the same temperature decrease; a higher temperature is required to fully expose the hydrophobic groups of the protein. In a 96-well Real-time Quantitative PCR (RT-PCR) plate, a solution was prepared with a final concentration of 1 mg/ml FadD23 protein, 9× SYPRO Orange Protein Gel Stain (Thermo Fisher Scientific, Waltham, MA, United States), 2 mM ATP, 2 mM MgCl_2_, and 2 mM fatty acid (octoic, decanoic, lauric, myristic, palmitic, and stearic acids) dissolved in 100% Dimethyl sulfoxide (DMSO) with a final well volume of 30 μl. The solution was allowed to equilibrate at 25°C for 20 min before a thermal melt protocol was initiated using a Bio-Rad CFX96 RT-PCR instrument (Bio-Rad Laboratories, Hercules, CA, United States). The “FRET” channel was selected to satisfy the excitation and emission wavelengths of SYPRO Orange (λex 470 nm/λem 570 nm). The melt sequence involved first maintaining the sample at 20°C for 5 min before linearly increasing the temperature by 1°C/min with fluorescence measurements every 30 s. Data were plotted as the derivative of the melting curve as a function of temperature.

### 2.7. Surface plasmon resonance

FadD23 was immobilized on a Series S Sensor Chip CM5 (Cytiva, Marlborough, MA, United States) to 3,000 response units (RUs) using a Biacore X100 (Cytiva, Marlborough, MA, United States) and running buffer composed of 10 mM PBS pH 7.4, 0.05% Tween 20, and 5% DMSO. Serial dilutions of fatty acid were injected at concentrations ranging from 100 to 0.19 mM. Response curves were fit to a 1:1 binding model using Biacore X100 Evaluation Software (Cytiva, Marlborough, MA, United States).

### 2.8. Hydroxamate-MesG assay

The 100-μl reaction system contained 20 mM Tris pH 8.0, 2 mM MgCl_2_, 1 mM DTT, 150 mM hydroxamate (pH 7.0), 0.1 U nucleoside phosphorylase (PNP), 0.04 U pyrophosphatase, 0.2 mM MesG, 1 mM ATP, 7.5 μM FadD23, and varying concentrations of fatty acids. Reactions were run in a 96-well plate (Corning 3,635; Corning Life Sciences, Corning, NY, United States) and monitored at A_360_ using a Varioskan™ LUX multimode microplate reader (Thermo Fisher Scientific, Waltham, MA, United States). The kinetic parameter of the substrate was determined for FadD23 using the hydroxamate formation assay; the standard assay conditions, described above, were used. The initial rate was measured as a function of the substrate concentration to provide a saturation curve, which was fit by nonlinear regression analysis to the Michaelis–Menten equation using GraphPad Prism 8.4. Then, *k*_cat_ was determined *k*_cat_ = *V*_max_/[*E*_t_]. All mutations or truncations determine initial rate under sufficient and consistent substrate conditions (100 μM).

## 3. Results

### 3.1. Overall structure of *Mycobacterium tuberculosis* FadD23

The oligomeric state of FadD23 is a monomer determined by size exclusion chromatography and further confirmed by analysis ultracentrifugation (AUC; Supplementary Figures S1A,B). Although many efforts have been directed toward the crystal screening test, apo-form *M. tb* FadD23 proteins failed to grow suitable crystals for data collection. However, well-shaped crystals from *M. tb* FadD23 with AMP-PNP or ATP could be diffracted to resolutions of 2.25 and 2.68 Å for data collection, respectively (Figure 1B). The full-length *M. tb* FadD23, containing a large N-terminal domain (1–460) and a small C-terminal domain (473–584), connect by flexible loops and an anti-parallel β-sheet (465–472), was obtained. *Mycobacterium tuberculosis* FadD23 has 14 helices, 23 β-sheets, and some irregular loops (Figure 1C). The N-terminal can be divided into three regions: regions A and B containing more than three helices and three sheets, and region C containing more flexible loops (Figures 1C,D). Regions A and B form two continuous sheets; sheet 1 comprising β1 and β2 form region A and β9, β10, β11, β12, and β13 form region B (Figures 1C,D, Supplementary Figure S2A). Sheet 2 consists of β3, β4, β5, and β6 to form region A and β7 and β8 to form region B (Figures 1C,D; Supplementary Figure S2B). In addition, region B surrounds a cavity for substrate binding. The C-terminal domain comprises three peripheral helices and three inner sheets (Figure 1C; Supplementary Figure S2C).

**Figure 2:**
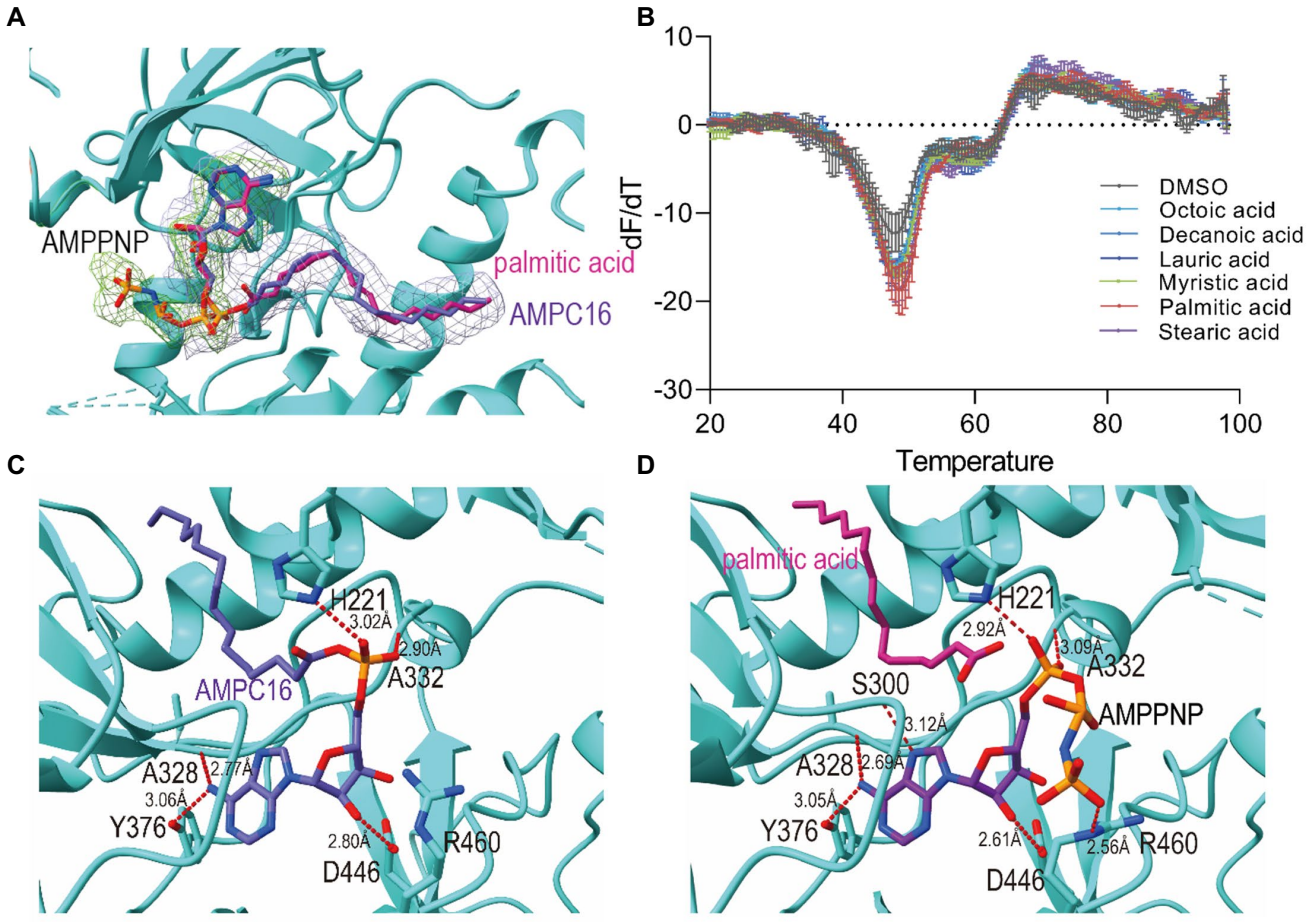
Active site and binding interactions of FadD23. **(A)** Ligand density for the ATP-FadD23 complex and AMP-PNP-FadD23 complex. OMIT maps were generated by removing the ligands, followed by a refinement cycle. The maps of the coefficients *F*_o_–*F*_c_ were contoured at 3.0σ. AMP-PNP: lime, hexadecanoyl adenylate (AMP-C16): silver. **(B)** Differential scanning fluorimetry analysis of FadD23 with fatty acid of different lengths. **(C)** Stereo representation of the hydrogen bonds (colored red) in ATP-FadD23 complex. **(D)** Arg460 and Ser300 each formed a hydrogen bond with AMP-PNP.

It was observed that two *M. tb* FadD23 complexes have extra electron densities in difference maps (*F*_o_–*F*_c_) near ATP or AMP-PNP (Figure 2A). After corroboration with previous studies and homologous structures (Zhang et al., 2011), it was concluded that this position is a substrate binding pocket, and its density indicates that it may be a fatty acid. The molecular weight of the small molecule obtained after protein denaturation and extraction was 256.4 Da, consistent with the size of palmitic acid (Supplementary Figure S1C). We surmised that it is derived from the *E. coli* expression system and binds to *M. tb* FadD23. Therefore, we interpreted these two densities as palmitic acid and hexadecanoyl adenylate (AMP-C16); the statistics and final refinement results are shown in Table 1. AMP-PNP is a non-hydrolysable ATP analog. FadD23 catalyzed the hydrolysis of ATP and connected AMP with palmitic acid to generate a molecule of AMP-C16. The structure of the ATP-FadD23 complex is similar to that of the AMP-PNP complex, with an RMSD of 0.2869 Å. In the structures of both complexes, no obvious electron density was observed for several loop regions, including residues 97–100, 125–137, 154–157, 172–176, and 511–513. However, a weak electron density was observed for parts of a loop (residue 553–557). The substrate is mainly located in the N-terminal domain, while ATP or AMP-PNP is bound to the contact surface between the N-(regions B and C) and C-terminal domains, and the fatty acid part points to the β-sheet of the N-terminal domain.

**Table 1:** Crystallographic and refinement statistics of FadD23 complexes and FadD23 N-terminal domain.

	ATP-FadD23 (8HD4)	AMP-PNP-FadD23 (8HDF)	FadD23 N-terminal domain (8HCZ)
Wavelength (Å)	0.97915	0.97853	0.97853
Cell parameters(Å)(˚)	73.90; 73.90; 464.0090; 90; 120	73.10; 73.10; 459.2090; 90; 120	67.64; 73.27; 91.7990; 90; 90
Space group	P6_1_22	P6_1_22	P2_1_2_1_2_1_
Completeness (%)	99.8 (99.2)a	99.9(99.7)	97.8(95.4)
Rmerge (%) b	11.4(106)	5.8(82.3)	18.5(41.5)
I/σ	13.70 (2.22)	22.35 (2.63)	7.97(3.63)
Resolution (Å)	50.0–2.68 (2.85–2.68)	50.0–2.24 (2.38–2.24)	50.0–1.48 (1.57–1.48)
No. of unique reflections	22117	36228	72121
No. of reflections in test set	1121	1803	3650
R_work_ (%)^c^	0.2095	0.2025	0.1769
R_free_ (%)^c^	0.2537	0.2500	0.1920
**rmsd from ideal values**			
Bond length (Å)	0.010	0.013	0.007
Bond angle (°)	1.226	1.252	0.943
Ramachandran outliers (%)	0	0.19	0.23

^a^Highest-resolution shell is shown parentheses. ^b^Rmerge=, where is an individual intensity measurement and is an average intensity for all reflections. ^c^R_work_/R_free_=, where and are the observed and calculated structure factors, respectively.

### 3.2. Binding affinity of *Mycobacterium tuberculosis* FadD23 with substrates

We suggested that palmitic acid in the structure was bound in the *M. tb* FadD23 hydrophobic pocket. Since *M. tb* FadD23 can obtain saturated fatty acids from the expression system, it is speculated that the protein can bind fatty acids. Therefore, the affinity of fatty acids of different lengths was determined to infer the range of carbon chain lengths with better binding capacity.

First, we use DSF to determine protein stability after adding different substrates. To verify our conjecture, we conducted experiments on FadD23 by adding different fatty acids, ATP, and Mg^2+^. After data processing, we observed that adding palmitic acid to FadD23 slightly increased the melting temperatures (*T*_m_; by approximately 0.5°C), indicating enhanced protein stability (Figure 2B, Supplementary Figure S3B). In addition, the *T*_m_ increased by 10°C after adding fatty acid, ATP, and Mg^2+^, further demonstrating enhanced stability (Supplementary Figures S3A,B). Furthermore, the protein had increased rigidity after substrate addition, which was conducive to acquiring protein crystals. Figure 2B shows the results as two valleys when ATP is absent, which can be regarded as two melting states (Figure 2B, Supplementary Figure S3A). When considering the addition of ATP, an apparent difference was observed regarding the importance of adenylation for stabilizing protein conformation.

To further determine the fatty acid specificity of FadD23, we measured the affinity of different fatty acids using a surface plasmon resonance system (Biacore T200; GE Healthcare, Chicago, IL, United States). The affinity of ATP to FadD23 reached 1 μM, and palmitic acid to FadD23 reached 3.450 × 10^−5^ M (Supplementary Table S2). At the same time, the binding ability between the protein and fatty acids increased with carbon chain length up to palmitic acid (the binding affinity increased); thereafter, increased carbon chain length weakened affinity. This provided robust evidence to speculate that palmitic acid is the optimal substrate for FadD23.

After various enzyme activity testing methods were conducted, the hydroxamate formation assay of Wilson and Aldrich (2010) was finally selected. In the absence of an acceptor, the tightly bound acyl-adenylate can be release of pyrophosphate (PPi) by the enzyme. The pyrophosphate produced is hydrolyzed to inorganic phosphate by pyrophosphatase that will react with MesG allowing the reaction to be monitored in a continuous fashion at 360 nm. We calculated the enzymatic activity of *M. tb* FadD23 under different substrate conditions. The *K*_m_, *k*_cat_, and *k*_cat_/*K*_m_ value for lauric acid are 33.89 μM, 2.40 × 10^−3^ s^−1^, and 71.8 s^−1^ mol^−1^ L, respectively. The *K*_m_, *k*_cat_, and *k*_cat_/*K*_m_ value for palmitic acid is 14.26 μM, 6.72 × 10^−4^ s^−1^, and 49.4 s^−1^ mol^−1^ L, respectively. However, the *K*_m_ value could not be fitted well with octanoic or stearic acid as substrates. It worth noting that the lower *K*_m_ value was that palmitic acid as substrate, a higher *k*_cat_/*K*_m_ value was obtained with lauric acid as substrate (Table 2).

**Table 2:** Kinetic parameters of FadD23.

Substrate	*V*_max_ (nmol·s^−1^)	*K*_m_ (μmol·L^−1^)	*k*_cat_ (s^−1^)	*k*_cat_/*K*_m_ (s^−1^·mol^−1^·L)
Octoic acid	-	ND	-	-
Lauric acid	1.80 × 10^−2^ ± 2.21 × 10^−3^	33.56 ± 5.40	2.39 × 10^−3^e−3 ± 2.94 × 10^−4^	71.2
Palmitic acid	4.90 × 10^−3^ ± 1.94 × 10^−4^	13.32 ± 1.20	6.54 × 10^−4^ ± 2.61 × 10^−5^	49.1
Stearic acid	-	ND	-	-

Next, we compared the fatty acid binding tunnels with full-length structures with other FAALs in *Mycobacteria*, including *M. marinum* (*M. m*) FadD32, *M. smeg* FadD32, and *M. tb* FadD10 (Liu et al., 2013; Guillet et al., 2016). The linear distances between the distal end and the portal of their fatty acyl binding tunnels were 7 Å, 14 Å or longer, and 10.8 Å, respectively. Moreover, FadD23 had a fatty acid binding pocket 15.5 Å in length, indicating the specificity of different FAALs with fatty acids (Supplementary Figures S4A–D).

### 3.3. Substrate binding site of *Mycobacterium tuberculosis* FadD23

**Figure 3:**
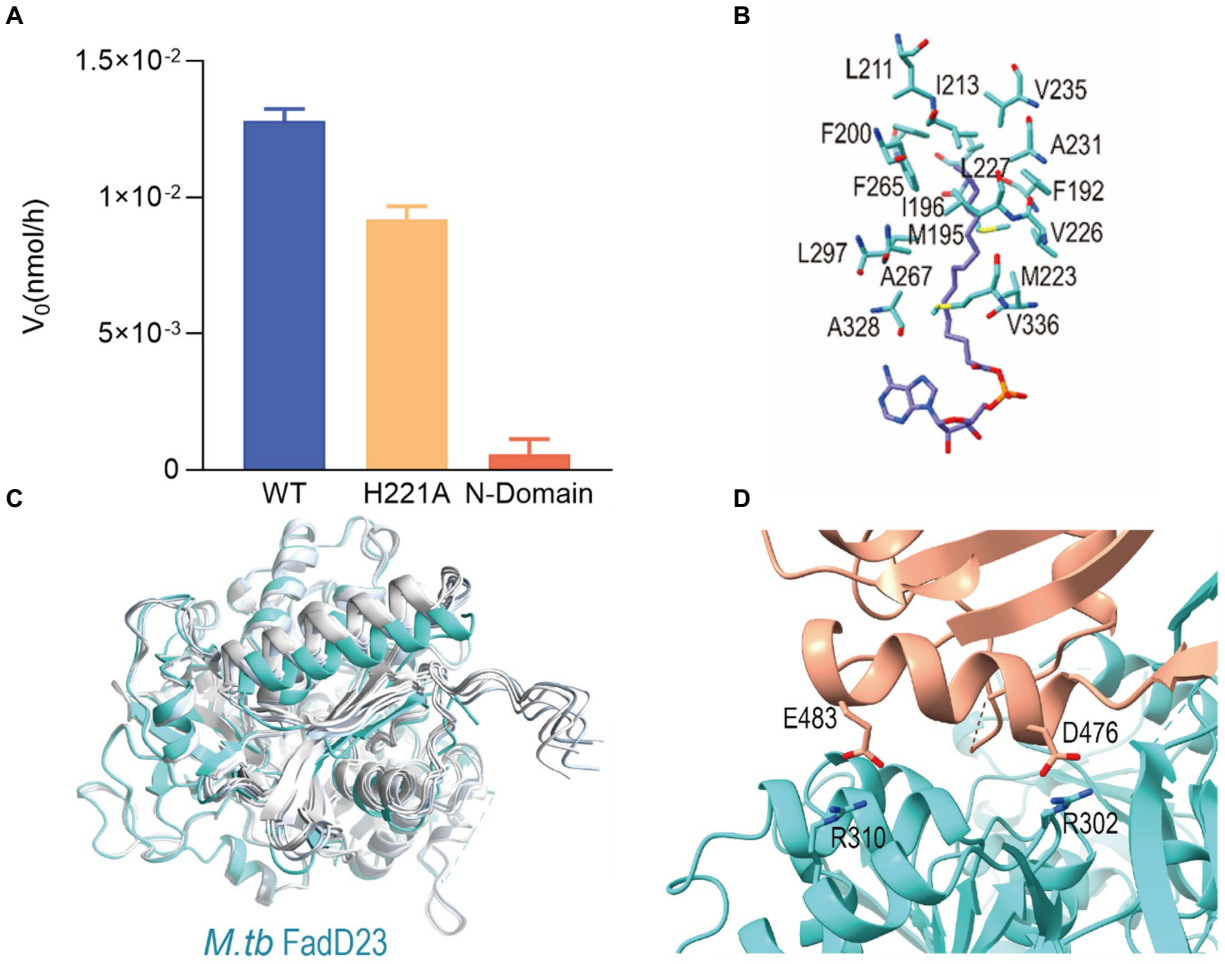
The key role of C-terminal domain of *Mycobacterium tuberculosis* (*M. tb*) FadD23. **(A)** The difference in the rate of enzyme activity of the mutants. **(B)** Stereo representation of the hydrophobic ATP-FadD23 complex. **(C)** Bottom view of the structure predicted by AlphaFold2 compared with that of FadD23. Blue and different shades of grey represent FadD23, Ranked_0, Ranked_1, Ranked_2, Ranked_3, and Ranked_4. **(D)** The interaction between the N- and C-terminal domains in the substrate-bound state.

It is evident in the crystal structure we obtained that the adenosine binding pocket is located in the cleft between the N-terminal and C-terminal domains and the residues involved in binding are all from the large N-terminal domain. The fatty acids or AMP-C16 were bound in the hydrophobic pocket of the N-terminal domain. Hydrogen bonds between FadD23 and adenosine groups were observed in the structure. The backbone oxygen of Ala328 and the -OH groups of Tyr376 formed two hydrogen bonds with N37 of adenosine. The NE2 group of His221 and the Nα of Ala332 were differentially bound to the monophosphate of AMP. The OD1 atom of Asp446 and O38 of the ribose group in adenosine formed a hydrogen bond (Figure 2C). All the mentioned interactions were also observed in the AMP-PNP-FadD23 complex (Figure 2D). At the same time, Asp222, Glu301, Arg302, Tyr329, Gly330, and Leu331 residues also interacted with AMP, contributing to the stability of the protein-substrate complex. In the AMP-PNP-FadD23 complex, the O27 of AMP-PNP interacted with NE of Arg460 through hydrogen bond, and Nα of Ser300 formed a hydrogen bond with N09 of AMP-PNP (Figure 2D). Based on these observed interactions, mutations were introduced. When His221 is replaced with Ala221 in FadD23, the activity of the enzyme was significantly reduced. The initial rate of the reaction was reduced to 75% of the original initial rate under sufficient and consistent substrate conditions (Figure 3A). We also compared AMP binding pockets of these structures. His221(*M. tb* FadD23) was conserved in FAALs. The backbone oxygen of Ala328 (*M. tb* FadD23) interacted with N37 of adenosine, Nα of Ala332 interacted with AMP, and the hydrogen bond between the OD1 atom of Asp446 (*M. tb* FadD23) and O38 of the ribose group were also observed in other FadDs (Supplementary Figure S4E).

Palmitic acid or hexadecanoyl adenylate was located in a hydrophobic pocket of the N-terminal domain. Met223, Ala328, and Val336 were located near the base of adenosine’s hydrophobic pocket, the amino acid residues in the middle of pocket were Met195, Tyr199, Val226, Leu227, and Leu297, while the amino acid residues Phe192, Ile196, Phe200, Leu211, Ala231, Val235, and Phe265 at the end of the pocket formed a semi-enclosed barrel (Figure 3B; Supplementary Figure S2D). The enveloping hydrophobic amino acid residues listed above constituted the binding pocket for the fatty acid substrate of FadD23. The length of hydrophobic pocket may correlate with substrate-specific selection.

### 3.4. C-terminal works as a “lid” for substrate binding

When in complex with a substrate, the C-terminal domain of *M. tb* FadD23 is structurally similar to the *M. smeg* FadD32. However, a large C-terminal domain shift presented in the *M. tb* FadD10-substrate structure can be observed compared to the structure of *M. tb* FadD23 complex with a substrate (Supplementary Figure S4F).

Based on the above results, we speculated that the C-terminal domain plays a key role in stabilizing the overall conformation. Next, we solved the 1.5 Å crystal structure of *M. tb* FadD23 N-terminal domain. The N-terminal structure was compared with that of the complex with ATP or AMP-PNP, and the RMSDs were observed to be 0.4265 and 0.3836 Å, respectively. No obvious changes in substrate binding sites were observed. At the same time, after analyzing the structure, no extra differential density was observed, indicating that the N-terminus of FadD23 could not capture fatty acids from the same expression system (Supplementary Figure S5B).

We inferred that the C-terminal domain serves as a cover for fatty acid binding to FadD23 and is important for the function of FadD23. The protein of the *M. tb* FadD23 N-terminal domain has no fatty AMP-ligase activity (Figure 3A).

Next, we used AlphaFold2 software to predict the structure of *M. tb* FadD23 and obtained five models with high confidence to compare structures by structurally aligning with the FadD23 (Figure 3C). The calculated RMSD values of structures, Ranked_0, Ranked_1, Ranked_2, Ranked_3, and Ranked_4, were 1.34, 1.02, 1.41, 1.43, and 1.13 Å, respectively. At the same time, the pLDDT of each residue was calculated (Supplementary Figure S6). It can be seen that among the five predicted structures, the confidence of the residues in the N-terminal domain was higher than that of the residues in the C-terminal domain. There are some low-confidence residues in both domains. In the structures we got, these residues are disordered, e.g., residues 96–100, or be stabilized when form complex with substrates, e.g., residues 552–556. Therefore, we speculated that these amino acid residues promote the occurrence of enzymatic activities in capturing substrates and generating products. Further, we speculated that the C-terminus resembles a lid that changes from a swinging (open) state to a stable (closed) state interacting with the N-terminal domain and the substrate after the substrate is bound to protein. Additionally, the anti-β-sheet (465–472) stabilizes the overall structure in the closed conformation.

Evaluation using PDBePISA^1^ revealed that the size of the interaction area between the two domains was 891.8 Å^2^. At the interface of the two domains, in addition to the interaction of the β-sheet, Arg310 and Glu483, Arg302, and Asp476 exhibited two hydrogen bonding interactions (Figure 3D).

### 3.5. Cross-talk between *Mycobacterium tuberculosis* FadD23 and Pks2

When *M. tb* FadD23 was expressed by the *M. smegmatis*, we observed the lanes of the peak one fraction eluted from gel chromatography in SDS-PAGE gel, in addition to that of FadD23, which had an obvious band at the position of approximately 220 kDa (Supplementary Figure S7). After verification using mass spectrometry, we confirmed that this is MSMEG_4727, which is homologous to Pks2 in *M. smegmatis* (data not shown).

## 4. Discussion

Previous research proved that *M. tb* FadD23 can transfer hexadecanoyl adenylate or AMP-C18 to Pks2 *in vivo* (Sirakova et al., 2001). We speculate the FadD23 binds to medium- or long-chain fatty acids (C8–C18). In this study, we verified the biological properties of FadD23 using different methods. We successfully detected the m/z corresponding to palmitic acid in the MS analysis. Despite the ample data generated by the enzymatic assay and MS analysis, we did not find sufficient evidence that could strongly support the binding selectivity of FadD23 to a specific long-chain fatty acid. Our research did, however, reveal that FadD23 directly captures palmitic acid from the *E. coli* expression system and that the palmitic acid-FadD23 binding slightly increases the *T*_m_.

We speculate that the reason for the two *T*_m_ values in DSF is that the first *T*_m_ peak is due to C-terminal domain instability, and the second one is caused by overall protein denaturation, which is also related to the conformational instability of the N- and C-terminals. The enzyme activity assay methods we initially used yielded no results. We either used a malachite green reagent to form a stable dark green color with the free phosphate released by the enzyme or a method determining the remaining amount of ATP that can be used by luciferase. We speculate that after the product was generated, since there was no downstream related protein to receive the product in time, the product could not be released. The hydroxamate formation assay, which depends on the ability of hydroxylamine to release tightly bound acyl-adenylate, was finally utilized. To explore the effect of changing the size of the hydrophobic pocket on the length of the bound fatty acid chain, we attempted to construct F192S, M195S, and F265S mutants, and we also introduced the S300A and D446A mutations according to structure analyzing or structure-based sequence alignments (Robert and Gouet, 2014; Supplementary Figures S4F, S5A) in FAALs. Unfortunately, no obvious protein expression was detected. Therefore, only FadD23 with H221A mutation could be used for subsequent enzymatic experiments. His221 is one residue that attached to the oxygen atom of α-phosphate both before and after AMP-C16 formation, gets closed (3.9 Å) to carboxyl group of the fatty acid. The histidine is conserved in FAALs according structure-based sequence alignments (Supplementary Figures S4F, S5A) in FAALs. That indicate that His221 should play a central role in hydrolysing ATP and producing the products. The reduction in enzymatic activity by 25% correlates with to hints provided by analyzing the interactions in structures and structure-based sequence alignments.

The crystal structures of FadD23 with either substrate, substrate analog, and N-terminal domain were obtained, but we did not obtain the structure of the apo state. Through the aforementioned structural analysis and biological properties studies, we infer that the mode of action of FadD23 includes the N-terminal domain binding to the substrate and the C-terminal domain functioning similarly to a lid that stably binds the substrate in the pocket to generate the product, which is then transported by downstream proteins. It is worth noting that the hydroxamate formation assay revealed that the binding capacity is nearly abolished when the C-terminal domain is absent. This is consistent with our hypothesis that the C-terminal domain plays a significant role in stabilizing substrate binding in the enzyme pocket.

**Figure 4:**
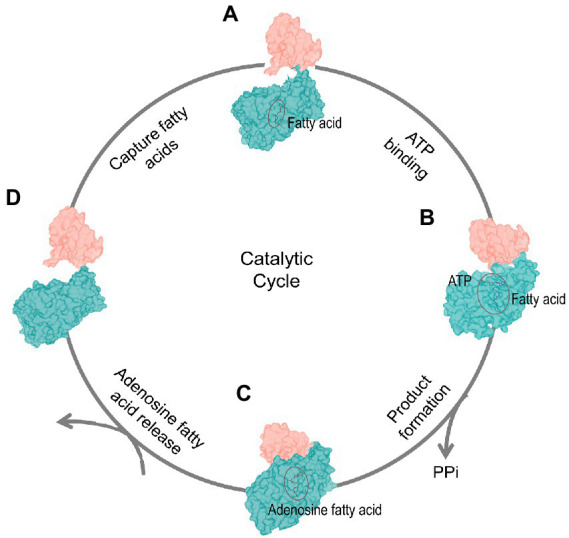
Dynamics of the catalytic cycle. The four-stage catalytic cycle of FadD23 activity. FadD23 captures fatty acids *in vivo* (Stage **A**). The binding of ATP induces structural changes (the C-terminal domain rotates, Stage **B**). The proximity of the N-(dark cyan) and C-(salmon) terminal domains promote the production of products (Stage **C**), and products are released (stage **D**).

Previous study took small angle X-ray scattering (SAXS) to elucidate the conformation changes of *M. smeg* FadD32 during catalysis process (Guillet et al., 2016). Because we did not obtain the structure of the apo state of FadD23, we took AlphaFold2 to predict the apo state of FadD23. The crystal structure of FadD23 with a substrate compared to the sequence-based predicted structure by AlphaFold2 can provide some hints for the conformational change in the catalytic process. We also speculate that the four-step catalytic cycle of FadD23 depends on analyzing all three structures and predictions from AlphaFold2 (Figure 4). FadD23 can capture fatty acids from the surrounding environment. Firstly, the C-terminal domain rotates after binding ATP and induces the conformation change in the full structure. Secondly, ATP de-pyrophosphate combines with palmitic acid to form a product (hexadecanoyl adenylate) that dissociates from FadD23 with the participation of downstream proteins. Finally, FadD23 returns to the apo state and waits for substrate capture. That speculation also consistent with research of *M. smeg* FadD32 (Guillet et al., 2016), and is a complementary understanding for *M. tb* FadD23. Moreover, we intended to verify the unloading of FadD23 products *in vitro* and clarify the relationship with downstream proteins, but we did not obtain any. This might be because more components are involved *in vivo*, which needs further exploration. It seems that FAALs transfer the acyl chain to the Pks modified with the P-pant arm through a special protein–protein interaction (Gavalda et al., 2009). We confirmed that FadD23 and Pks2 could be co-purified when over-expression FadD23 in *M. smegmatis*. However, it is unclear whether the transfer of the substrate requires the participation of other proteins. Therefore, the structural basis of the interaction between FadD23 and Pks2 or other proteins and the mode of action between these proteins need to be elucidated in the future.

Previous studies have mentioned that substrate analogs, such as tetradecyl-AMS for *M. tb* FadD28 or PhU-AMS for *M. tb* FadD32, could be treated as general inhibitors for different FAALs. *In vitro* anti-tubercular activity experiments proved that PhU-AMS inhibits *M. tuberculosis* with a minimum inhibitory concentration 3.13 μM (Kuhn et al., 2016; Baran et al., 2020). We also attempted to demonstrate the inhibition of the enzymatic activity of *M. tb* FadD23 using PhU-AMS, but PhU-AMS has no inhibitory activity against FadD23 (data not shown). As *M. tb* FadD23 and *M. tb* FadD28 have 66% sequence similarity and share the same evolutionary branch, it is very interesting that PhU-AMS have no effect on enzymatic activity of *M. tb* FadD23. Therefore, we speculate that PhU-AMS has no inhibitory effect on FadD23 because it considerably differs from its optimal substrate as the FAAL class of FadDs appears to functional nonredundant in production defer fatty acids (Sourjik and Berg, 2004). In a word, identifying a specific FadD23 inhibitor will be very complicated.

In summary, we elucidated the structure of full-length *M. tb* FadD23, the first protein in the SL-1 synthesis pathway. Through structural, biological, and chemical analyses, we postulate that palmitic acid is the preferred biological substrate of FadD23. Based on our results, we believe that the FadD23 N-terminal domain alone cannot efficiently bind fatty acids without C-terminal domain and the C-terminal domain plays a crucial role in the catalytic mechanism. However, more studies are needed to clearly elucidate the mechanism of SL-1 synthesis pathway and there provides a theoretical background for designing FadD23 inhibitors. Whether a specific inhibitor could reduce SL-1 production with the possible effect of attenuating virulence could do reducing the bacterial load in the host is still a question, more efforts are needed to develop specific inhibitors against FAALs or selective inhibitors of FadDs.

## Data availability statement

The data presented in the study are deposited in the RCSB Protein Data Bank (www.rcsb.org), accession numbers 8HD4, 8HDF, 8HCZ.

## Author contributions

M.Y., W.Z. (Wei Zhang) and Z.R. conceived the study. M.Y., W.Z. (Wei Zhang), L.C., L.Z. performed the experiments. M.Y., L.C., W.Z. (WeiHong Zhou) and W.Z. (Wei Zhang) analyzed the data. M.Y., X.L. and W.Z. (Wei Zhang) wrote the paper. W.Z. (Wei Zhang) and Z.R. supervised this project.

## Funding

This work was supported by the National Key Research and Development Program of China (Grant No. 2017YFC0840300), the National Natural Science Foundation of China (Grant Nos. 81520108019 to ZR, 32100142 to WZ, and 32171259 to XL), and the Fundamental Research Funds for the Central Universities, Nankai University (Grant Nos. 63181333, 63191431, and 63201095 to XL).

## Conflict of interest

The authors declare that the research was conducted in the absence of any commercial or financial relationships that could be construed as a potential conflict of interest.

## Publisher’s note

All claims expressed in this article are solely those of the authors and do not necessarily represent those of their affiliated organizations, or those of the publisher, the editors and the reviewers. Any product that may be evaluated in this article, or claim that may be made by its manufacturer, is not guaranteed or endorsed by the publisher.

## Footnotes

^1^ https://www.ebi.ac.uk/msd-srv/prot_int/cgi-bin/piserver
