# Supplementary material for: The Key Roles of Mycobacterium tuberculosis FadD23 C-terminal Domain in Catalytic Mechanisms
Source: Front Microbiol. 2023 Feb 21;14:1090534. doi: 10.3389/fmicb.2023.1090534 (PMC9989471; doi:10.3389/fmicb.2023.1090534)
Supplement: Supplementary file 1 [file Data_Sheet_1.pdf]

Supplementary Material

Supplementary Material

1 Supplementary Figures and Tables

1.1 Supplementary Figures

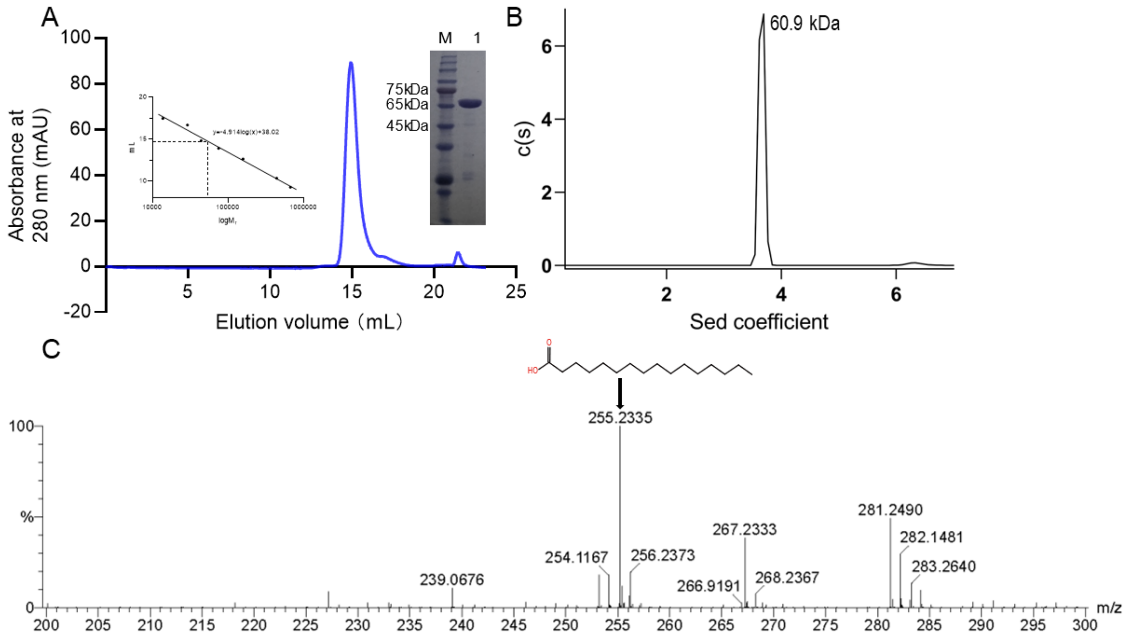

Supplementary Figure 1. FadD23 purification results and characterization analysis. (A) Recombinant FadD23 protein was purified by Ni-NTA affinity chromatography followed by size exclusion chromatography. The peak fraction at 14.7 mL in superdex200 increase 10/300 GL, corresponding to a molecular mass between 44 000 Da (Ovalbumin) to 75 000 Da (Conalbumin), and the purified FadD23 was analyzed by SDS-PAGE. The single band corresponding to a molecular mass of FadD23 appeared in lane 1. (B) Analytical ultracentrifugation of FadD23 exogenously expressed in *E. coli*. (C) The presence of palmitic acid in the purified protein was detected by mass spectrometry.

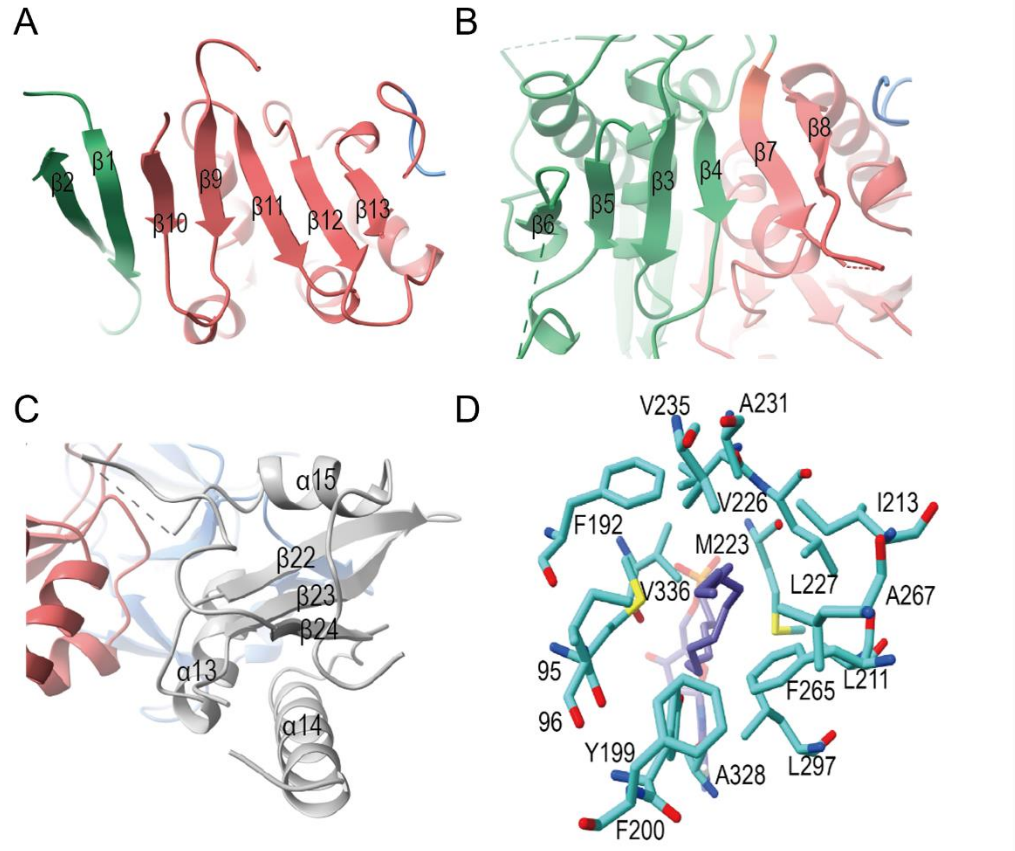

Supplementary Figure 2. Interaction analysis in crystal structures of ATP-FadD23 complex. (A) Sheet 1 comprising β1 and β2 from region A, and β9, β10, β11, β12, and β13 from region B. (B) Sheet 2 comprising β3, β4, β5, and β6 from region A, and β7 and β8 from region B. (C) The FadD23 C-terminal domain is mainly composed of three peripheral helices and three inner strands. (D) Stereo representation of the hydrophobic ATP-FadD23 complex.

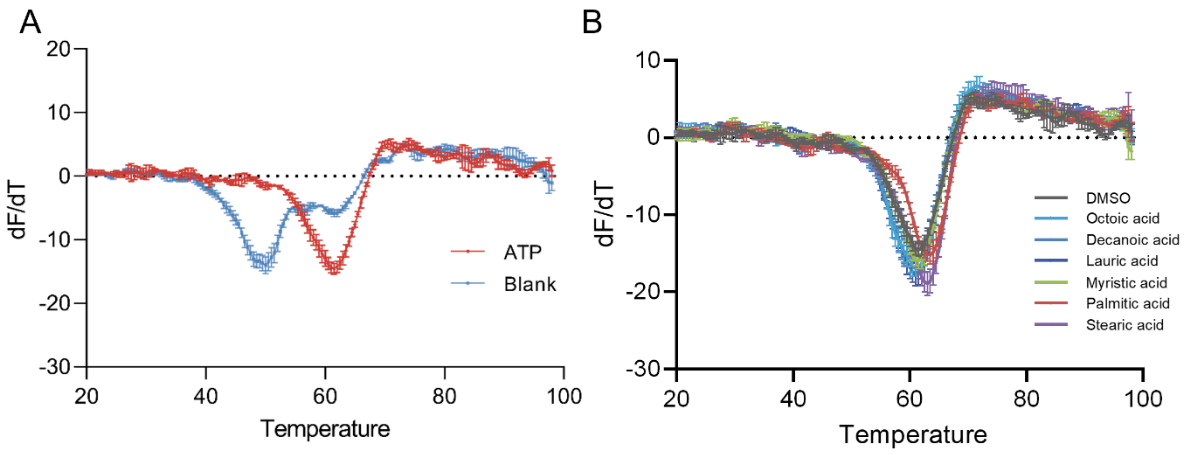

Supplementary Figure 3. Differences in protein stability of FadD23 in different states. (A) Differential scanning fluorimetry analysis of FadD23 with or without ATP. (B) Differential scanning fluorimetry analysis of FadD23 and ATP with fatty acids of different lengths.

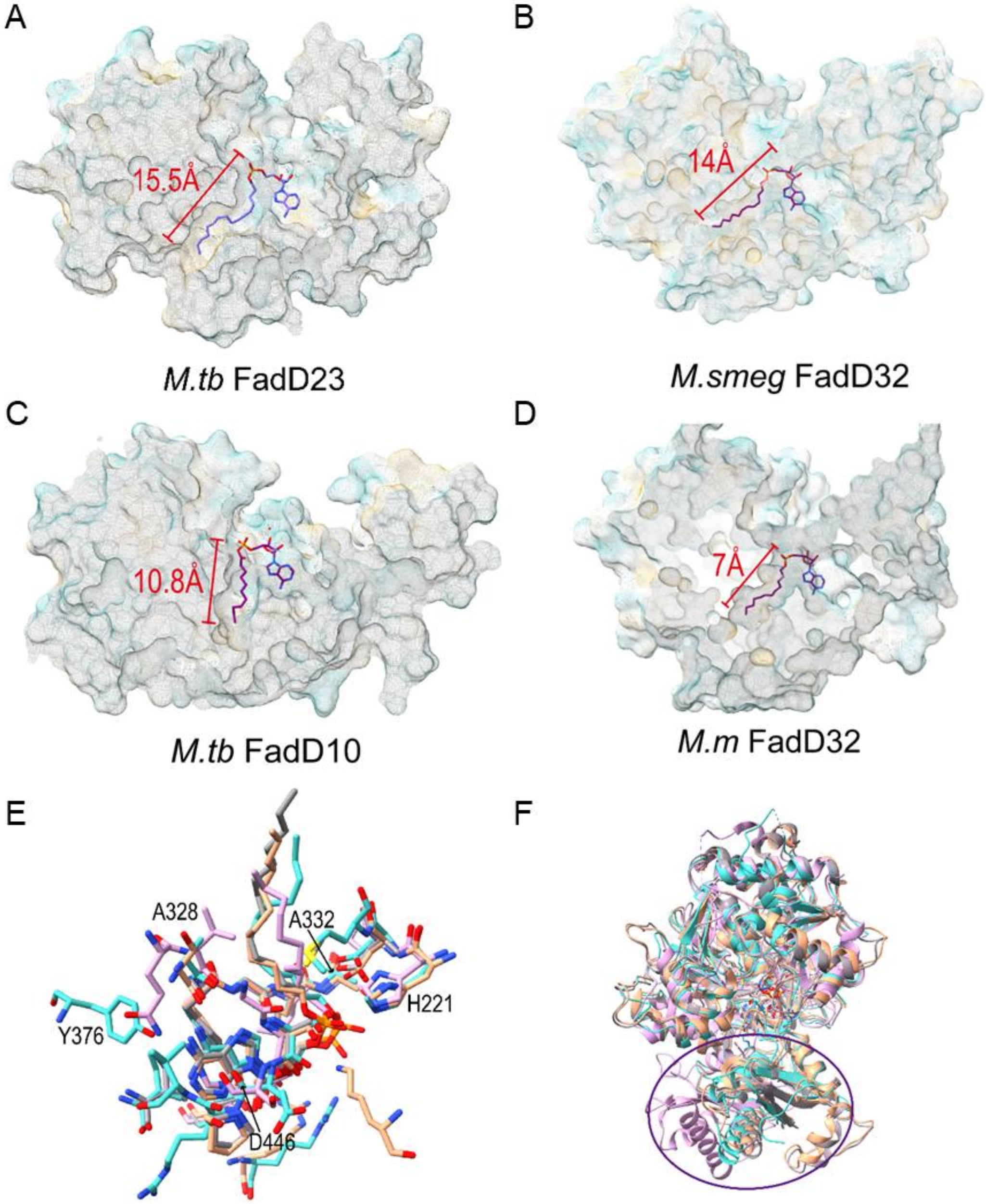

Supplementary Figure 4. Comparison of the substrate-binding pockets of the mycobacterial FadD family. Fatty acid-AMP colored purple in each crystal structure. (A) *M. tb* FadD23 with hexadecanoyl adenylate. (B) *M. m* FadD32 with AMPC12 (PDB code:5EY9). (C) *M. tb* FadD10 with AMPC12 (PDB code:4IR7). (D) *M. smeg* FadD32 with AMPC12 (PDB code: 5EY8). (E) Comparison of active site of substrate pocket. *M. tb* FadD23 colored in mediumturquoise, *M. smeg* FadD32 colored in chatoyant, *M. m* FadD32 colored in light salmon, *M. tb* FadD10 colored in plum. (F) C-terminal domains diversity in different FadDs. *M. tb* FadD23 colored in mediumturquoise, *M. smeg* FadD32 colored in chatoyant, *M. m* FadD32 colored in light salmon, *M. tb* FadD10 colored in plum.

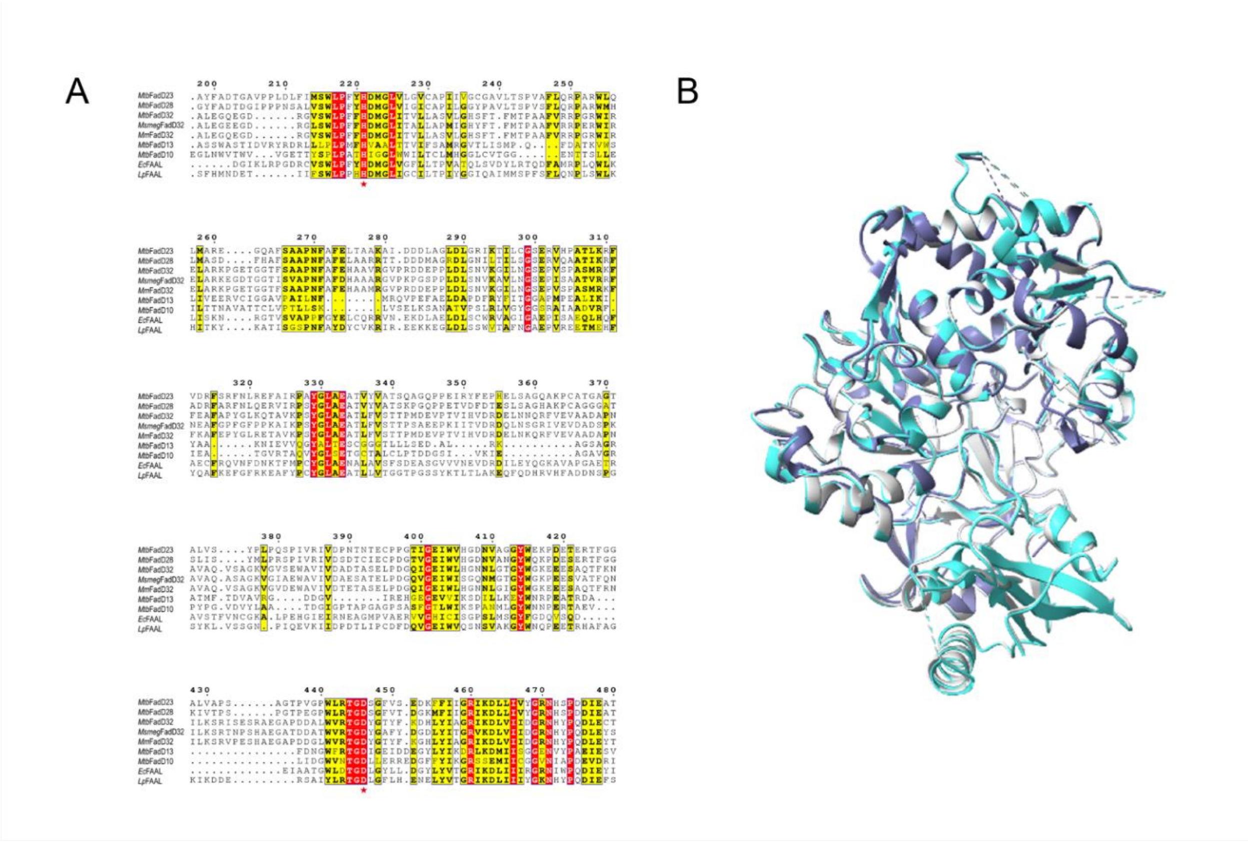

Supplementary Figure 5. Sequence and structure alignment of FadD23. (A) Sequence alignment of M. tb FadD23 with another FAALs. (B) Front view of structure of ATP-FadD23 complex, AMP-PNP-FadD23 complex and FadD23 N-terminal domain. ATP-FadD23 complex represented in mediumturquoise, AMP-PNP-FadD23 complex represented in light grey, FadD23 N-terminal domain represented in dark blue-gray, respectively.

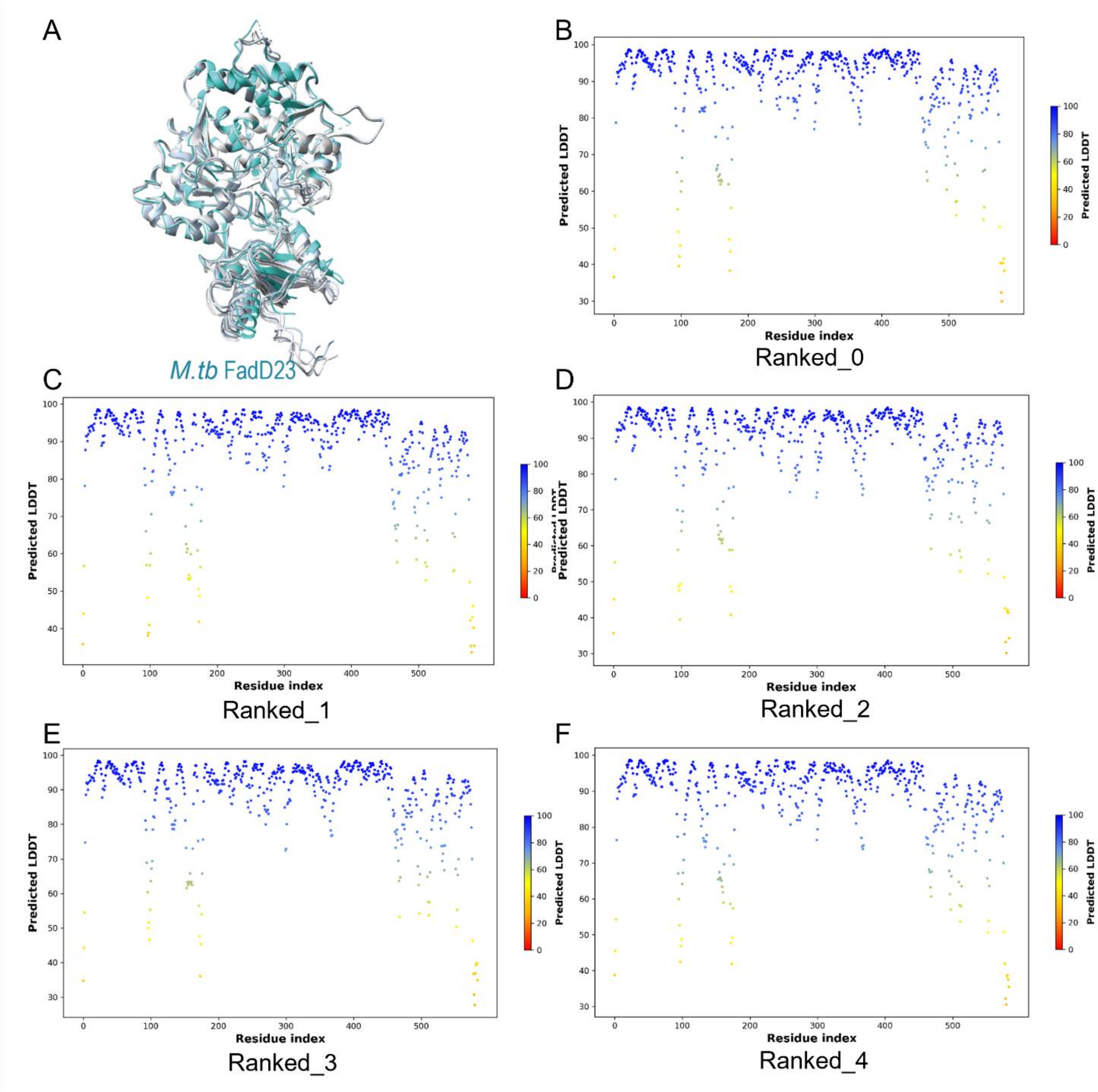

Supplementary Figure 6. AlphaFold2 analysis results. (A) Front view of the structure predicted by AlphaFold2 compared with that of FadD23. FadD23, Ranked_0, Ranked_1, Ranked_2, Ranked_3, and Ranked_4 are represented in blue and different shades of grey. (B), (C), (D), (E), and (F) show the pLDDT of predicted structures.

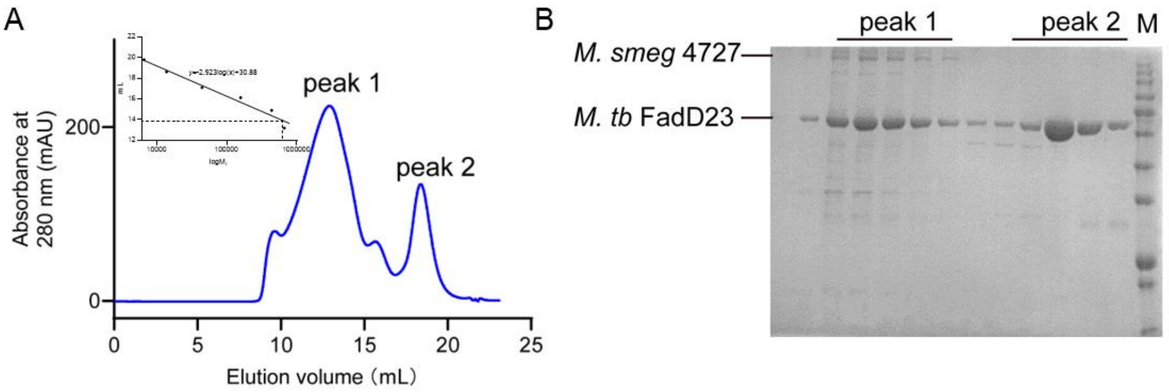

Supplementary Figure 7. The preliminary result of M.tb FadD23 purified using the *Mycobacterium smegmatis* mc^2-155 expression system. (A) Gel chromatography (column: superose 6 increase 10/300 GL) of purification. There are two major peaks labeled as peak 1 and peak 2. (B) Polyacrylamide gel (12%) electrophoresis analysis of FadD23 gel chromatography purification. Lanes under the lines of peak 1 and peak 2 correspond to fractions of peak 1 and peak 2 of gel chromatography purification.

1.2 Supplementary Tables

Supplementary Table 1. Primers used in the study

Primer name	Primer sequence
pET15_FadD23_IF	CGCGGCAGCCATATGATGGTTTCGCTTTCC
pET15_FadD23_IR	GCAGCCGGATCCTTAAAGATCGCTCGCTTG
pET15_FadD23N_IF	CGCGGCAGCCATATGATGGTTTCGCTTTCC
pET15_FadD23N_IR	CTTTGTTAGCAGCCGGATCCTTACAACAGATCCTTTATTC
23_A231W_F	GGGTTTGGTTCTGGGAGTTTGTTGGCCGATTATCGTAG
23_A231W_R	CCAACAAACTCCCAGAACCAAACCCATGTCATGATAG
23_A328W_R	CCAGGGCCGAATTGCGAATTCTCGAAGATTGAAACGGC
23_D446A_IF	GCCTTGGCTACGAACTGGCGCCTCGGGCTTC
23_D446A_IR	GGCGCCAGTTCGTAGCCAAGGCCCTACGGGTG
23_F192SM195S_IF	GAATATATTGGCAAATAGCCAGCAGAGCATTTCCGCCTATTTCGC
23_F192SM195S_IR	GCGAAATAGGCGGAAATGCTCTGCTGGCTATTTGCCAATATATTC
23_F265S_IF	CACGCGAGGGCCAGGCGAGCTCGGCGGCACC
23_F265S_IR	GCTCGCCTGGCCCTCGCGTGCCATCAATTGCAGC
23_G330W_F	CGCAATTCGGCCCGCGTACTGGCTCGCGGAAG
23_G330W_R	CCAGTACGCGGGCCGAATTGCGAATTCTCGAAG
23_H221A_F	GTCGTGGCTACCGTTCTATGCCGACATGGGTTTGG
23_H221A_R	GGCATAGAACGGTAGCCACGACATAATGAAAAGGTCC
23_S300A_IF	CAAAACCATCCTCTGCGGCGCTGAAAGGGTGC
23_S300A_IR	AGCGCCGCAGAGGATGGTTTTGATCCGTCCAAGG
pET15_FadD23c_IF	GCCGCGCGGCAGCCATATGTTGATTGTTTACGGCCG
pmv261-FadD23-IF	CTTCCAATCCAATGCTATGGTTTCGCTTTCC
pmv261-FadD23-IR	TATCCCACCCAAATGAAGATCGCTCGCTTG

Supplementary Table 2. Binding affinity of FadD23 (SPR).

Substrate	K_D (M)
ATP	1.03×10^{-6}
Octoic acid	6.906×10^{-5}
Lauric acid	4.495×10^{-5}
Palmitic acid	3.450×10^{-5}
Stearic acid	ND
